# Learning the Randleman Criteria in Refractive Surgery: Utilizing ChatGPT-3.5 Versus Internet Search Engine

**DOI:** 10.7759/cureus.64768

**Published:** 2024-07-17

**Authors:** Jared J Tuttle, Majid Moshirfar, James Garcia, Amal W Altaf, Soroush Omidvarnia, Phillip C Hoopes

**Affiliations:** 1 Ophthalmology, University of Texas Health Science Center at San Antonio, San Antonio, USA; 2 Hoopes Vision Research Center, Hoopes Vision, Draper, USA; 3 John A. Moran Eye Center, University of Utah School of Medicine, Salt Lake City, USA; 4 Eye Banking and Corneal Transplantation, Utah Lions Eye Bank, Murray, USA; 5 Medicine, University of Arizona College of Medicine – Phoenix, Phoenix, USA; 6 Medicine, Texas Tech University Health Sciences Center, El Paso, USA

**Keywords:** post-lasik ectasia, lasik, keratoconus, corneal and refractive surgery, artificial intelligence and education, chatgpt-3.5, prompt engineering, randleman criteria, medical education, self-directed learning (sdl)

## Abstract

Introduction

Large language models such as OpenAI's (San Francisco, CA) ChatGPT-3.5 hold immense potential to augment self-directed learning in medicine, but concerns have risen regarding its accuracy in specialized fields. This study compares ChatGPT-3.5 with an internet search engine in their ability to define the Randleman criteria and its five parameters within a self-directed learning environment.

Methods

Twenty-three medical students gathered information on the Randleman criteria. Each student was allocated 10 minutes to interact with ChatGPT-3.5, followed by 10 minutes to search the internet independently. Each ChatGPT-3.5 conversation, student summary, and internet reference were subsequently analyzed for accuracy, efficiency, and reliability.

Results

ChatGPT-3.5 provided the correct definition for 26.1% of students (6/23, 95% CI: 12.3% to 46.8%), while an independent internet search resulted in sources containing the correct definition for 100% of students (23/23, 95% CI: 87.5% to 100%, p = 0.0001). ChatGPT-3.5 incorrectly identified the Randleman criteria as a corneal ectasia staging system for 17.4% of students (4/23), fabricated a “Randleman syndrome” for 4.3% of students (1/23), and gave no definition for 52.2% of students (12/23). When a definition was given (47.8%, 11/23), a median of two of the five correct parameters was provided along with a median of two additional falsified parameters.

Conclusion

Internet search engine outperformed ChatGPT-3.5 in providing accurate and reliable information on the Randleman criteria. ChatGPT-3.5 gave false information, required excessive prompting, and propagated misunderstandings. Learners should exercise discernment when using ChatGPT-3.5. Future initiatives should evaluate the implementation of prompt engineering and updated large-language models.

## Introduction

Keeping up with the rapidly advancing field of refractive surgery can prove to be a challenge for entry-level learners. Residents and medical students hoping to engage in the field must identify reliable sources of information for self-directed learning [1]. The future of refractive surgery depends upon the ability to identify accurate, reliable, and efficient learning tools to educate the next generation of refractive surgeons.

Recent advances in artificial intelligence (AI) have introduced new avenues for medical education. An AI-powered large language model (LLM) can consolidate vast swaths of information into a singular, interactive chatbot [2]. Functioning as a personal tutor, an LLM can respond to follow-up questions, rephrase concepts, and generate unlimited practice cases. These novel capacities suggest that LLMs hold promise in augmenting self-directed medical learning. Therefore, steps must be taken to ensure that information provided by an LLM is accurate, reliable, and efficient within a self-directed learning environment.

In November 2022, OpenAI (San Francisco, CA) released Chat Generative Pre-Trained Transformer version 3.5 (ChatGPT-3.5), a publicly available LLM trained on an extensive dataset sourced from the internet [3]. ChatGPT-3.5 has demonstrated expertise across diverse medical specialties and performed near the passing threshold for all three parts of the United States Medical Licensing Exam (USMLE) [4-8]. However, concerns regarding accuracy have surfaced in highly specialized areas of medicine [9-12].

The present study compares the performance of ChatGPT-3.5 and an internet search engine in providing information on the Randleman criteria. First introduced in 2008, the Randleman criteria represent a model for identifying patients at high risk of developing corneal ectasia following laser in situ keratomileusis (LASIK) surgery [13-17]. Its five parameters include corneal topography, residual bed thickness, patient age, corneal thickness, and preoperative manifest refraction spherical equivalent (MRSE). As a well-defined, thoroughly discussed topic in refractive surgery, the Randleman criteria present an ideal challenge for ChatGPT-3.5.

The study’s primary outcome is the ability of ChatGPT-3.5 or an internet search engine to define the Randleman criteria and its five parameters within a self-directed learning environment. In addition, a qualitative analysis of the ChatGPT-3.5 conversation threads is provided, yielding valuable insights for learners of refractive surgery.

## Materials and methods

Data collection

Twenty-three medical students participated in the study between May 2023 and December 2023. Participation was restricted to currently enrolled medical students with an expressed interest in ophthalmology and no prior knowledge of the Randleman criteria. Each author collaborated with the Hoopes Vision Research Center and recruited eligible participants from their respective institutions.

Participants were informed that the study aimed to assess the effectiveness of two different learning tools: ChatGPT-3.5 and an internet search engine. To mimic a self-directed learning situation, the topic “the Randleman criteria in ophthalmology” was provided with no additional contextual information.

Each participant was instructed to interact with ChatGPT-3.5 within a single conversation thread. Participants were allowed to use an unlimited number of prompts within a 10-minute time limit. Participants were not allowed to search the internet during the conversation with ChatGPT-3.5. Afterwards, participants were asked to submit a copy of the conversation thread and a summary of their findings.

Following the session with ChatGPT-3.5, participants were instructed to learn about the Randleman criteria using an internet search engine of their choice for a maximum duration of 10 minutes. Participants then provided a summary of their findings with a list of references.

Each entry was collected via e-mail and assigned an entry number between 1 and 23.

Data analysis

Each ChatGPT-3.5 conversation thread was examined, with the provided definition of the Randleman criteria and its five parameters being recorded, if any were given. Discrepancies from the definition and parameters described by Randleman et al. were noted [13]. Prompts given to ChatGPT-3.5 were counted and characterized. Innovative prompts were identified, including requests for mnemonic aids, tables, case examples, and comparative explanations. Instances where ChatGPT-3.5 requested more context, advised the student, or recognized a knowledge cut-off date were documented.

References discovered via internet search were categorized by publication type and inspected for accuracy. Each student’s reflective summary was evaluated and compared against the information found in their accompanying references.

The McNemar test was run using R software (R Foundation for Statistical Computing, Vienna, Austria) to compare the paired samples with dichotomous outcomes of incorrect and correct definitions of the Randleman criteria. Post-hoc power analysis suggests that a sample size of 23 yields a power of 0.92 with an alpha of 0.00001. Confidence intervals (CI) were calculated using the modified Wald method.

## Results

Primary outcomes: correct definition and parameters

**Figure 1:**
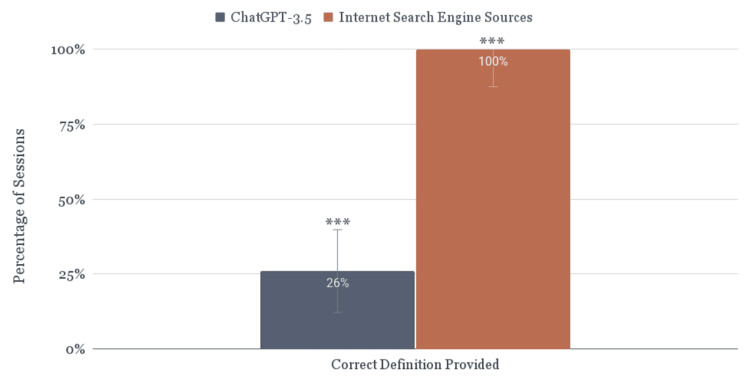
Comparing the ability to accurately define the Randleman criteria. *** indicates statistical significance (p = 0.0001) as calculated by the McNemar test of paired proportions.

**Figure 2:**
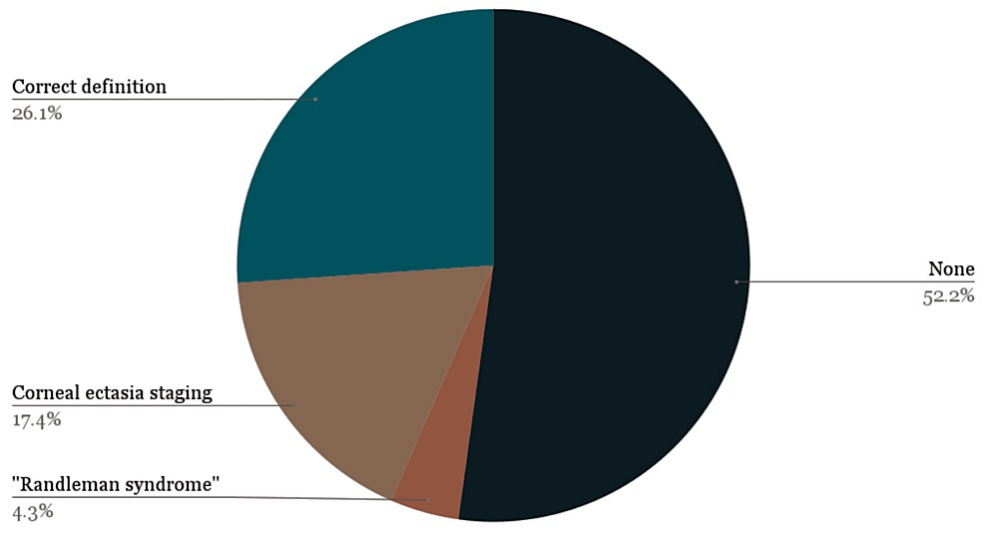
Definitions of the Randleman criteria provided by ChatGPT-3.5. Correct definition: ChatGPT-3.5 identified the Randleman criteria as a model used to predict the development of postoperative corneal ectasia. Corneal ectasia staging: ChatGPT-3.5 erroneously defined the Randleman criteria as a system to stage the development of corneal ectasia. “Randleman syndrome”: ChatGPT-3.5 fabricated a novel disease. None: ChatGPT-3.5 failed to provide a definition of the Randleman criteria.

**Figure 3:**
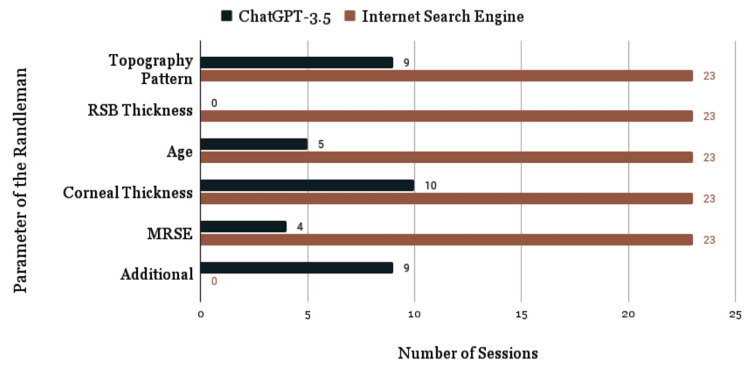
Parameters of the Randleman criteria provided by ChatGPT-3.5 and internet search engine. RSB: residual stromal bed; MRSE: preoperative manifest refraction spherical equivalent; Additional represents fabricated parameters of visual acuity (3), corneal curvature (2), pachymetry (2), and family history (2).

ChatGPT-3.5 provided the correct definition for 26.1% of students (6/23, 95% CI: 12.3% to 46.8%), while an independent internet search resulted in sources containing the correct definition for 100% of students (23/23, 95% CI: 87.5% to 100%, p = 0.0001, Figure 1). ChatGPT-3.5 incorrectly identified the Randleman criteria as a corneal ectasia staging system for 17.4% of students (4/23), fabricated a “Randleman syndrome” for 4.3% of students (1/23), and gave no usable definition for 52.2% of students (12/23, Figure 2). When a definition was provided (47.8%, 11/23), a median of two of the five correct parameters was provided along with a median of two additional falsified parameters (Figure 3). Corneal thickness (81.8%, 9/11) and corneal topography (72.7%, 8/11) were the most frequently identified parameters, while residual stromal bed thickness was never mentioned. The most commonly identified false parameters were visual acuity (27.3%, 3/11), corneal curvature (18.2%, 2/11), and family history (18.2%, 2/11). Table 1 displays the definitions and parameters provided in each ChatGPT-3.5 conversation thread and internet search.

**Table 1:** Results of each ChatGPT-3.5 conversation and internet search. * ChatGPT-3.5 initially supplied the listed definition; however, over the course of the conversation, the definition was retracted. ** Participant provided an incorrect definition of the Randleman criteria despite citing the original paper describing the criteria. N/A: non-applicable, parameters were not provided through ChatGPT-3.5; CT: preoperative corneal thickness; MRSE: preoperative manifest refraction spherical equivalent; VA: visual acuity; IOP: intraocular pressure; BCVA: best corrected visual acuity.

ID	ChatGPT-3.5					Internet search engine		
	Definition given	Number of correct parameters	Correct parameters	Number of additional false parameters	False parameters	Definition given	Number of correct parameters	Sources used
1	None	N/A	-	-	-	Correct**	5	[13-15]
2	"Randleman syndrome"	2	CT, topography	2	VA, IOP	Correct	5	[13,14]
3	Ectasia staging system	2	CT, topography	3	Corneal scarring, VA, contact lens tolerance	Correct	5	[12-14]
4	Ectasia staging system	2	CT, topography	1	VA	Correct	5	[13,14]
5	None	N/A	-	-	-	Correct	5	[14]
6	Correct	4	CT, topography, age, MRSE	0	-	Correct	5	[12,13]
7	Correct	4	CT, topography, age, MRSE	3	Corneal elasticity, surgical history, family history	Correct**	5	[13,14]
8	Ectasia staging system	2	CT, topography	1	BCVA	Correct	5	[13,15]
9	Ectasia staging system*	2	CT, topography	2	Corneal curvature, pachymetry	Correct	5	[14]
10	Correct	4	CT, topography, age, MRSE	4	Corneal curvature, pupil size, family history, others	Correct	5	[12,14]
11	Correct*	0	-	0	-	Correct	5	[12]
12	None	N/A	-	-	-	Correct	5	[12]
13	None	N/A	-	-	-	Correct	5	[16]
14	None	N/A	-	-	-	Correct	5	[14,15]
15	Correct	4	CT, topography, age, MRSE	2	Pachymetry, endothelial cell count	Correct	5	[13-15]
16	None	N/A	-	-	-	Correct	5	[13]
17	None	N/A	-	-	-	Correct	5	[12,13]
18	Correct	2	CT, age	5	Stable refraction, overall health, patient expectations, ocular disease, cornea health	Correct	5	[14]
19	None	N/A	-	-	-	Correct	5	[13,14]
20	None	N/A	-	-	-	Correct	5	[14]
21	None	N/A	-	-	-	Correct	5	[12,15]
22	None	N/A	-	-	-	Correct	5	[14]
23	None	N/A	-	-	-	Correct	5	[12]

Prompt analysis

ChatGPT-3.5 conversation threads contained a median of four total prompts, with a minimum of two and a maximum of 10 prompts. The majority of initial queries (78.2%, 18/23) did not provide any contextual information to ChatGPT-3.5, with the most common prompt (22.2%, 7/23) being “What is/are the Randleman criteria?”. The term “ophthalmology” was included in 21.7% (5/23) of initial prompts. Multi-sentence prompts were not utilized. Table 2 contains the initial query, access date, and total number of prompts for each entry.

**Table 2:** Prompt characterization for each ChatGPT-3.5 conversation. * ChatGPT-3.5 initially supplied the listed definition; however, over the course of the conversation, the definition was retracted. N/A: non-applicable – a definition was not provided by ChatGPT-3.5.

ID	Initial prompt provided to ChatGPT-3.5	Date of entry	Total number of prompts	Number of prompts to get a definition	Definition given
1	"What is the Radioman criteria"	5/24/23	10	N/A	None
2	"Explain what the Randleman criteria is"	5/24/23	4	N/A	"Randleman syndrome"
3	"What is the Randleman criteria in the field of ophthalmology?"	5/24/23	4	2	Ectasia staging system
4	"What is the Randleman criteria?	5/24/23	7	5	Ectasia staging system
5	"Identify what the Randleman criteria is.	5/24/23	4	N/A	None
6	"Tell me about the Randleman criteria in ophthalmology"	5/24/23	6	2	Correct
7	"What are the Randleman criteria?"	5/24/23	8	1	Correct
8	"Explain the Randleman criteria in a simple paragraph"	6/1/23	9	1	Ectasia staging system
9	"What is the Randleman criteria?"	6/1/23	7	2	Ectasia staging system*
10	"What are the Randleman criteria?"	6/1/23	9	2	Correct
11	"What is the Randleman criteria in the field of ophthalmology?"	7/18/23	10	1	Correct*
12	"Tell me about the Randleman criteria."	7/31/23	3	N/A	None
13	"Ophthalmology Randleman criteria"	8/3/23	4	N/A	None
14	"Randleman criteria"	8/2/23	2	N/A	None
15	"Teach me about the Randleman criteria"	8/1/23	5	2	Correct
16	"Can you tell me a little bit about the Randleman criteria?"	8/11/23	2	N/A	None
17	"What is the Randleman criteria"	8/15/23	2	N/A	None
18	"Explain the Randleman criteria please"	8/17/23	2	1	Correct
19	"Explain the Randleman criteria"	12/19/23	2	N/A	None
20	"What are the Randleman criteria?"	12/19/23	3	N/A	None
21	"Can you explain the Randleman criteria"	12/20/23	4	N/A	None
22	"Can you explain to me the Randleman criteria"	12/20/23	4	N/A	None
23	"Can you tell me about the Randleman criteria in regards to opthomology."	12/20/23	3	N/A	None

ChatGPT-3.5 was challenged to provide definitions, case applications, value cut-offs, tables, mnemonics, and comparisons to other clinical models. ChatGPT-3.5 successfully defined LASIK, criteria, ectasia, and corneal ectasia for students. When asked to apply the Randleman criteria to a clinical case (17.3%, 4/23 conversations), partially accurate examples were supplied in each case. ChatGPT-3.5 did not provide specific cut-off values for parameters when requested (13.0%, 3/23 conversations). When ChatGPT-3.5 was asked to compare the Randleman criteria to other criteria and classification systems (30.4%, 7/23 conversations), a response was provided for 71.4% of requests (5/7 requests), with partially accurate information being provided in each case. When directly prompted for a table (4.3%, 1/23 conversations), ChatGPT-3.5 successfully provided a summary of the Randleman criteria in tabulated format. In another instance (4.3%, 1/23 conversations), ChatGPT-3.5 provided a mnemonic to assist in remembering the criteria.

ChatGPT-3.5 directly requested additional context in 39.1% (9/23) of conversations, but the student responded with additional context in only two instances (22.2%, 2/9 requests). Notably, ChatGPT-3.5 was unable to interpret and correct a misspelled prompt (“Radioman” criteria) (4.3%, 1/23 conversations).

Secondary outcomes: efficiency, accuracy, and reliability

For students who received a usable definition (11/23), a median of two prompts was required to elicit a definition (min: 1; max: 5). Falsified information was present in every ChatGPT-3.5 conversation session (23/23). False information included inaccurate definitions of the criteria (5/11), incomplete criteria parameters (23/23), partially correct case applications (4/23), and incorrect names of researchers (3/23). In 78.3% of conversations (18/23), ChatGPT-3.5 identified a knowledge cut-off date and encouraged the student to refer to the most recent medical literature for up-to-date information.

When utilizing an independent internet search, student summaries referenced a median of three references. Of the students, 91.3% (21/23) referenced a peer-reviewed journal article, with 47.8% of students (11/23) citing the original article by Randleman et al. [13] and 69.6% of students (16/23) citing EyeWiki [15,17]. Two summaries (8.7%, 2/23) contained false information despite having referenced accurate sources from the internet.

## Discussion

Our study found that the internet search engine outperformed ChatGPT-3.5 in providing accurate and reliable information on the Randleman criteria. ChatGPT-3.5 gave false information, required excessive prompting, and propagated misunderstandings. Dangerously, two student summaries contained false information from ChatGPT-3.5 even after the students had consulted the correct internet sources. Thus, exposing students to inaccurate information may result in insidious misunderstandings that resist correction. Learners of refractive surgery must be made aware of the risks associated with using ChatGPT-3.5 as an independent learning tool.

A common criticism of LLMs has been their inability to indicate the source of provided information, ChatGPT-3.5 included. A surrogate for credibility is to reference the dataset used to train the LLM. We found that ChatGPT-3.5 consistently identified a knowledge cut-off date for its training data. However, when ChatGPT-3.5 failed to provide a definition for the Randleman criteria, students mistakenly assumed that the topic must have been introduced after the knowledge cut-off date. For an LLM to be consistently used as a learning tool, credibility and reliability must be established, a current pitfall of ChatGPT-3.5.

Since the release of ChatGPT-3.5, newer, more effective LLMs have already begun to surface. Released in March 2023, Google Bard has outperformed ChatGPT-3.5 in certain topics [18]. In addition, GPT-4, OpenAI’s successor to ChatGPT-3.5, has demonstrated superiority on subspecialty practice questions [14,19,20]. However, GPT-4 requires a paid subscription, limiting its accessibility to learners. Additional LLMs are in production and likely to emerge soon, including those trained on more reliable datasets [21]. Further evaluations will be necessary to ensure the validity of such learning tools.

As LLMs progress, the development of effective “prompt engineering” has the potential to augment learning efficiency [22]. Prompt engineering refers to the development of effective prompts with abundant context [23]. In our study, students failed to use multi-sentence prompts and inconsistently provided background information, even when requested directly by ChatGPT-3.5. While a scarcity of such information is expected for self-directed learners, training in prompt engineering may be a valuable investment, especially as LLMs improve.

This study was limited as ChatGPT-3.5 was queried on only one refractive surgery topic. ChatGPT-3.5’s performance in this study may not represent its expertise on all topics within refractive surgery. Furthermore, the efficacy of an LLM is dependent upon the prompts provided. The prompting capabilities of medical students in this study may not be representative of all medical students. Finally, learner comprehension was not assessed in this study. Future evaluations could assess learning outcomes when using an LLM or a combination of resources.

## Conclusions

Although LLMs hold promise in augmenting medical learning, findings from this study raise significant concerns regarding the accuracy of ChatGPT-3.5 in providing information on refractive surgery. In the self-directed learning environment where context is limited and efficiency is paramount, ChatGPT-3.5’s role as an independent resource is currently limited. Providing training in prompt engineering to medical learners may improve their ability to extract quality information from ChatGPT-3.5 and other LLMs. The development of more accurate, efficient, and reliable LLMs would prove to be an asset in advancing medical education. In the interim, these results suggest that learners in refractive surgery should exercise caution when using ChatGPT-3.5 as a self-directed learning tool.
